# Bio-psychosocial factor of vaginismus in Iranian women

**DOI:** 10.1186/s12978-021-01260-2

**Published:** 2021-10-18

**Authors:** Mojdeh Banaei, Nourossadat Kariman, Giti Ozgoli, Maliheh Nasiri

**Affiliations:** 1 Student Research Committee, Department of Midwifery and Reproductive Health, School of Nursing and Midwifery, Shahid Beheshti University of Medical Sciences, Tehran, Iran; 2 Midwifery and Reproductive Health Research Center, School of Nursing and Midwifery, Shahid Beheshti University of Medical Sciences, Tehran, Iran; 3 Department of Biostatistics, School of Nursing and Midwifery, Shahid Beheshti University of Medical Sciences, Tehran, Iran

**Keywords:** Vaginismus, Sexual dysfunction, Bio-psychosocial model, Islamic Republic of Iran, Women

## Abstract

**Background and aim:**

Various physical, psychological, social and cultural factors contribute to vaginismus. Therefore, given the multidimensionality of this disorder and the need to pay more attention to all biological, psychological and social dimensions in its treatment, the present study was conducted to investigate the bio-psychological factors contributing to vaginismus.

**Methods:**

This descriptive cross-sectional study was conducted on 180 Iranian women with vaginismus who had been referred to sexual health clinics of Tehran province in 2020. Multistage random sampling method was used in this study, and vaginismus was diagnosed in women by a specialist through using a questionnaire. Data collection tools included demographic and obstetric information form, valid and reliable Sexual Function Questionnaire, Depression Anxiety Stress Scales (DASS), Sex Fear Questionnaire, Vaginal Penetration Cognition Questionnaire, Sexual Self-Efficacy Scale, Sexual Knowledge and Attitude Scale, Sexual Quality of Life-Female, Inventory of Sexual Satisfaction, ENRICH Marital Satisfaction Scale, Sexual Intimacy Scale and Questionnaire for Diagnosis of Vaginismus. In order to determine the factors related to vaginismus, multiple linear regression model was used through SPSS software version 25 (SPSS Inc., Chicago, IL).

**Results:**

Based on the results of the present study, the mean age of women and the mean duration of their marriage were 27.77 ± 5.36 and 4.07 ± 3.87 years respectively. As the results of multiple linear regression revealed, the variables of fear of sex (B = 0.141, P = 0.036), positive cognition (B = 0.197, P = 0.046), self-image (B = 0.651, P = 0.001), sexual intimacy (B = -0.116, P = 0.021), quality of sexual life (B = 0.115, P = 0.002) and education (B = 2.129, P = 0.024) from the bio-psychosocial model were the final predictors of vaginismus diagnosis score in women with this disorder. According to the results of multiple linear regression, 45.5% of the variance of vaginismus diagnosis total score was explained by these variables (R = 0.706, R2 = 0.498 and ADJ.R2 = 0.455).

**Conclusion:**

The results of the present study showed that the variables of fear of sex, positive cognition and negative self-image, sexual intimacy, quality of sexual life and education were the final predictors of vaginismus diagnosis score. This disorder is, thus, considered to be multidimensional.

## Plain language summary

As a sexual dysfunction, Vaginismus prevents sexual penetration through involuntary and frequent spasms of the muscles in the one-third of vagina's outer part**.** Factors such as negative beliefs about sex, cultural factors, fear of pain, injury, bleeding and so forth play significant roles in the prediction of vaginismus. Therefore, given the multidimensionality of vaginismus and the need to pay attention to all biological, psychological and social dimensions in treating it, and since the bio-psychosocial model is a strong framework for the factors contributing to sexual problems whose recognition will lead to the design of multidimensional treatments, the present study was conducted on 180 Iranian women with vaginismus to investigate the bio-psychological factors associated with vaginismus. The present study showed that the fear of sex, positive cognition and negative self-image, sexual intimacy, quality of sexual life and education from the bio-psychosocial model were the final predictors of vaginismus diagnosis score. Therefore, this model can be used in designing interventions for the treatment of vaginismus, especially in the psychological and interpersonal domains.

## Background

Sexual health is an essential aspect of personal health that may affect all people in all ages and stages of life [1]. It includes a complex and ambiguous combination of physical, emotional, psychological and social dimensions of health and is exposed to many challenges and issues [2]. Sexual function includes desire, arousal, orgasm, and suppression, and is affected by many bio-psychosocial factors [3]. Sexual dysfunction, on the other hand, refers to a series of psychosexual disorders and unpleasant experiences of individuals and couples which show themselves as impaired sexual desire, sexual arousal, orgasm and pain during sexual intercourse [4]. Vaginismus is a sexual dysfunction that prevents sexual penetration via involuntary and frequent spasm of the muscles in the one-third of vagina's outer part [5].

Based on previous studies conducted on women admitted to sexual health clinics, the prevalence of this disorder has been 7.8% in Italy [6] and 6.4% in Portugal [7]. In a study in Iran, about 33% of Iranian women expressed their experience of pain or fear during intercourse [8]. According to recent evidence, the incidence of vaginismus is higher in young women, women with sexual abuse, and women with negative attitudes towards sex. This disorder is a culture-dependent disorder and has been reported more in Ireland, Eastern Europe and Latin America than in North America and Western Europe [9].

Factors such as family history, negative beliefs about sex, cultural factors such as valuing the hymen [10], fear of pain, injury, bleeding, fear of losing one's control and having a panic attack [5] play significant roles in the prediction of vaginismus. As shown by recent studies, vaginismus is more prevalent among educated women with high socioeconomic status. Strong religious beliefs and feelings of guilt about intercourse can contribute to this disorder [11]. Women with vaginismus have more concerns about losing control, negative body image, catastrophic pain, and beliefs about their genital incompatibility during vaginal penetration. These women have also less knowledge and positive beliefs about vaginal penetration [12, 13]. The less the negative cognition of pain and the more the positive cognition of vaginal penetration, the better will be the couple's sexual satisfaction and function [14].

Many sexual problems of couples are caused by the lack of knowledge and experience, sexual misconceptions and inability of couples to express their sexual interests and priorities [15]. Ellison has stated that vaginismus is primarily due to the lack of sexual knowledge and guilt about sex, both of which lead to fear of sex [16]. In general, women with vaginismus have negative views about their gender and premarital sex [17, 18].

This disorder can lead to physical pain, depression, and feelings of frustration and sexual dysfunction, which may gradually cause problems in the sexual desire of most women with this disorder [19]. These problems may also be associated with other sexual disorders such as sexual arousal disorder and dissatisfaction with sexual life [20]. In societies where premarital relationships are forbidden, vaginismus has a tremendous negative effect on the sexual life of couples and is often a serious threat to the family foundation [21]. In this regard, Binik and Meana believe that all bio-psychosocial factors should be considered in the treatment of sexual dysfunction [22].

Based on recent evidence, the bio-psychosocial model is a key element in standard sexual medicine care. The bio-psychosocial model integrates the biological, psychological, and social aspects of patients and their health into an inseparable framework [23]. Making an interaction between biological, psychological, and social factors, this model determines the cause, manifestation, and outcome of wellness and disease [24]. The bio-psychosocial approach states that all causes and consequences of sexual disorders include a combination of these factors and aims to provide a comprehensive treatment that incorporates the biological, psychological and social aspects of a sexual disorder [25].

Therefore, given the multidimensionality of vaginismus and the need to pay attention to all biological, psychological and social dimensions in its treatment, and since the bio-psychosocial model is a strong framework for the factors contributing to sexual problems whose recognition will lead to the design of multidimensional treatments, the present study aimed to investigate the bio-psychological factors associated with vaginismus.

## Materials and methods

### Participants, procedure

As a descriptive cross-sectional study, the present study was conducted on 180 women with vaginismus from April 2020 to November 2020 in Tehran, Iran. Multistage sampling method was used in this study. In order to select the sampling locations, first, the sexual health clinics of Tehran province were divided into four categories of north, south, east and west, and from each category, the clinics with the most clients were selected. Then, the sample size in each clinic was determined by quota method and considering the number of the clients (number of sample in northern clinic = 35, southern clinic = 65, eastern clinic = 45 and western clinic = 40) and, finally, sampling was performed in the clinics by using convenience sampling method. Then, in each clinic the samples were selected in such a way that they were the representatives of the community as much as possible and were appropriate to the research objectives. Using statistical formula (type I error 0.05 and test power 0.9) and the study of Fadul et al. [5], the sample size of the study was estimated to be 180 subjects.

Inclusion criteria consisted of the 18–45-years-old women who were Iranian, literate, single spouse, and had the experience of at least six months of sexual activity, history of pain during intercourse or inability to have intercourse because of spasm and contraction, response to the examination in the form of pain and spasm/contraction, avoidance behaviors, definitive diagnosis of primary vaginismus by a vaginismus diagnostic questionnaire and its confirmation by a specialist. Moreover, the criteria for not including the subjects in the study were having sexual dysfunction in the spouse based on the person's statements, having serious family conflicts in the last month based on the wife's statements, pregnancy, breastfeeding and menopause, chronic medical illness, experience of a stressful event in the last three months, drug addiction, mental illness, and the use of psychotropic drugs that affect sexual desire. Exclusion criteria also included not answering more than 5% of the questionnaire's questions (Except Questionnaire for Diagnosis of Vaginismus, answering to its questions is necessary for diagnosing vaginismus).

This study was approved by the ethics committee of Shahid Beheshti University of Medical Sciences. The researcher referred to the centers and after explaining the objectives of the study to the women, a written consent form was completed for them if they were willing to participate in the study and have the inclusion criteria. The participants were assured that their information will be kept confidential. Vaginismus was diagnosed in the participants based on the vaginismus diagnostic questionnaire and then by a specialist based on DSM-IV criteria. Then, the study questionnaires were completed by the participants.

### Main outcome measure

Data collection tools in this study included demographic and obstetric information form, Sexual Function Questionnaire, Depression Anxiety Stress Scales (DASS), Sex Fear Questionnaire, Vaginal Penetration Cognition Questionnaire, Sexual Self-Efficacy Scale, Sexual Knowledge and Attitude Scale, Sexual Quality of Life-Female, Inventory of Sexual Satisfaction, ENRICH Marital Satisfaction Scale, Sexual Intimacy Scale and Questionnaire for Diagnosis of Vaginismus.

#### Female Sexual Function Index

Female sexual function index is a 19-item questionnaire for measuring women's sexual performance in six areas of sexual function including sexual desire, sexual arousal, vaginal moisture, orgasm, satisfaction, and pain during sexual activity. These subcategories have a response range of 1 to 5, and a score above 5 indicates a better sexual function. The total score of each person is equal to the sum of the individual's scores in each area, which is a maximum score of 36 [26]. Fakhri et al. confirmed the reliability of this tool by two methods of internal agreement and test retest (ICC = 0.77; α = 0.86) [27]. In the present study, to measure reliability, Cronbach's alpha coefficient of sexual function was calculated to be 0.931.

#### Depression, Anxiety, Stress Scale (DASS)

This questionnaire was used by Lovibond et al. in 1995 as a 42-item questionnaire to measure depression, anxiety and stress. Its shortened form has 21 questions where 7 questions are allocated to each feeling of depression, anxiety and stress, and is graded based on a 4-point scale from 0 to 3. The range of scores in each area is between 0 and 21 [28]. The validity and reliability of the Persian version of this scale was assessed by Samani and Jokar [29]. To measure the reliability of this scale in the present study, Cronbach's alpha coefficient of depression, anxiety and stress was calculated to be 0.898, 0.752 and 0.782 respectively.

#### Sex Fear Questionnaire

The frequency of women's experience of fear in different sexual situations was measured by the Dutch version of the Sex Fear Questionnaire. Five items of this questionnaire are about fear of sex without penetration and three items about fear of sex with penetration. Answers to the questions of this questionnaire are based on a 5-point Likert scale. Cronbach's alpha coefficient for the subscales of fear of sex with and without penetration was 0.73 and 0.71 respectively [30]. To measure reliability in the present study, Cronbach's alpha coefficient of fear of sex was calculated to be 0.853.

#### Vaginal Penetration Cognition Questionnaire

The initial version of this questionnaire had 40 questions that, after initial validity and reliability by Klaassen and Ter Kuile, were reduced to 22 questions. This tool is to examine the thoughts and feelings of women with vaginismus and dyspareunia. This tool measures five factors related to the cognition of vaginal penetration [12]. In the present study, after obtaining permission from the tool designer by the researcher and using the forward–backward method, the relevant tool was translated into Persian. Then, face, content and construct validities of the tool were determined by exploratory and confirmatory factor analysis on 352 eligible women. In exploratory factor analysis, three factors (Catastrophic and control cognition, Positive cognition and Self-image cognition) were extracted for the Persian version of VPCQ which explained 53.945% of the variance of the VPCQ variables and, using the confirmatory factor analysis, the 3-factor model was approved. Then, the reliability of the questionnaire was determined by the test–retest method and also by examining the internal correlation of the items. Cronbach’s alpha and maximum reliability of all factors were acceptable. The average size of ICC was 0.995. The absolute reliability of the tool was confirmed with SEM and MDC estimations of 2.67 and 0.28 respectively.

#### Sexual self-efficacy

Sexual self-efficacy scale was developed by Vaziri and Lotfi Kashani in 2013 based on Schwarzer's general self-efficacy scale. The prepared questionnaire has 10 questions which have been scored based on a 4-point scale ranging from 0 to 3. The range of scores is between 0 and 30 and a higher score means a higher level of self-efficacy [31]. The validity and reliability of this tool were evaluated by Vaziri and Lotfi Kashani in 2013. The reliability of the questionnaire was calculated to be 0.865 using Cronbach's alpha coefficient and 0.817 using the bisection method [31]. To measure reliability, Cronbach's alpha coefficient of sexual self-efficacy was calculated to be 0.894 in the present study.

#### Sexual Knowledge and Attitude Scale

This 30-item scale was designed and psychometrized by Besharat in 2005 to measure the two dimensions of knowledge and sexual attitude in Iranian society. The tool measures sexual knowledge and attitude based on a 5-point Likert scale which is scored from 1 to 5. The minimum and maximum scores of the questionnaire are 30 and 150 respectively. To measure the reliability of the questionnaire, the internal consistency was determined to be appropriate through calculating Cronbach's alpha coefficient of 0.91 and 0.88 for the subscales of knowledge and sexual attitude respectively [32]. To measure reliability, Cronbach's alpha coefficient of knowledge and sexual attitude was calculated to be 0.881 in the present study.

#### Sexual quality of life-female

This scale was developed by Symonds et al. in 2005. This scale has 18 items with a 6-point Likert scale ranging from strongly agree to strongly disagree and a higher score means a higher quality of sexual life [33]. The tool has adapted to the Iranian society by Masoumi et al. and the internal consistency of it has been confirmed using Cronbach's alpha index of 0.73 [34]. To measure reliability, Cronbach's alpha coefficient of sexual quality of life was calculated to be 0.80 in the present study.

#### Inventory of sexual satisfaction

The inventory of sexual satisfaction was designed by Hudson et al. in 1981. Answers to the 25 items of this tool are scored based on a 5-point Likert scale. The minimum and maximum scores are 25 and 125 respectively [35]. The validity and reliability of the tool in Iran were confirmed by Bahrami et al. in 2016. Cronbach's alpha for positive sexual satisfaction questions was 0.80 and the reliability of negative sexual satisfaction questions was 0.77, and the intra-cluster correlation coefficient was calculated to be 0.80 [36]. For the measurement of reliability in the present study, Cronbach's alpha coefficient of sexual satisfaction was calculated to be 0.781.

#### ENRICH Marital Satisfaction Scale

This scale was designed and psychometrized by Fower et al. in 1993 to measure marital satisfaction. The validity and reliability of the 10-item version of this questionnaire have been evaluated in Iran. Using the Likert scale, the questions of the scale are given a score of 1 to 5 and, therefore, the higher the score, the more satisfactory is the married life [37, 38]. The validity and reliability of the questionnaire in Iran was confirmed by Alidosti et al. in 2015. The reliability of the questionnaire was estimated to be 0.74 using Cronbach's alpha coefficient [38]. To measure reliability, Cronbach's alpha coefficient of marital satisfaction was calculated to be 0.753 in the present study.

#### Sexual Intimacy Scale

Botlani et al. prepared the sexual intimacy scale based on authoritative scientific sources, Bagarozi's Sexual Intimacy Questionnaire and the studies conducted in this area. This 30-item questionnaire examines the needs of couples with regard to intimacy and the emotional, psychological, intellectual, sexual, physical, spiritual, aesthetic and social-recreational dimensions. Each question is answered based on a 4-point scale. The minimum and maximum scores are 30 and 120 respectively, and the higher the score, the better is sexual intimacy [39]. Using Cronbach's alpha, the reliability coefficient of this questionnaire was confirmed by Shakarmi et al. (2014) to be 0.78 [40]. To measure reliability, Cronbach's alpha coefficient of sexual intimacy was calculated to be 0.927 in this study.

#### Questionnaire for diagnosis of vaginismus

This two-part questionnaire was designed and psychometrized by Raisi et al. in 2015. The first part of the questionnaire is related to the therapist and is completed after the gynecological examination based on the indicators of pain, spasm and avoidance of the examination. The second part includes 17 questions which are answered by patients in four areas (the area of physical suffering and inability to have sex, the area of inappropriate feeling in sexual intercourse, the area of inappropriate background conditions in sexual intercourse and the area of fear and lack of desire for sex). The overall cut-off point of the patient-related part is 54 [41]. To measure reliability, Cronbach's alpha coefficient of the questionnaire for the diagnosis of vaginismus was calculated to be 0.761 in the present study.

### Statistical analysis

The extracted data were statistically analyzed using SPSS software version 25 (SPSS Inc., Chicago, IL). Descriptive statistics were used to calculate percentage, mean and standard deviation, and inferential statistics to analyze and find relationships. First, the distribution of quantitative variables was investigated using Kolmogorov–Smirnov tests, and in case of normal distribution, parametric statistical tests and otherwise non-parametric tests were used. In order to determine the factors related to vaginismus, multiple linear regression with inter approach was used; so that, first univariate regression was performed where the dependent variable was the score of the questionnaire for the diagnosis of vaginismus and independent variables were the variables with a lower than 0.5 significance level (P < 0.5), which entered the initial model of multiple linear regression. Variance inflation factor (VIF) was used to evaluate Multicollinearity.

## Results

Generally, 180 women with vaginismus participated in the present study. Table 1 shows the demographic and sexual information of the participants. 69.4% of women stated that they always have foreplay before intercourse. On average, they had 7.43 ± 5.15 times unsuccessful vaginal penetration each month, in most cases they had man-on-top position when trying for penetration (57.8%).

**Table 1:** Socio-demographic and sexual information of 180 Iranian women with vaginismus

Socio-demographic variables		M ± SD/n-%	Sexual information variables		M ± SD/n-%
Age (years)		27.77 ± 5.36	Number of unsuccessful penetration (per month)		7.43 ± 5.15
Age of partner (years)		31.50 ± 3.25	Frequency of foreplay	Always	125 (69.4)
				Often	51 (28.3)
				Never	4 (2.2)
Duration of relationship (years)		4.07 ± 3.87	Start being sexual relationship	Women	7 (3.9)
				Men	96 (53.3)
				Both	77 (42.8)
Education	Academic	126 (70)	Having anal sex	Yes	34 (18.9)
	Non-academic	54 (30)		No	146 (81.1)
Education of partner	Academic	105 (58.3)	History of sexual assults	Yes	30 (16.7)
	Non-academic	75 (41.7)		No	150 (83.3)
Employment	Housewife	127 (70)	Having masturbation	Yes	83 (46.1)
	Employee	53 (29.4)		No	97 (53.9)
Marriage pattern	Traditional	96 (53.3)	Position of attempted penetration	Superior men	104 (57.8)
	Modern	84 (46.7)		Superior women	14 (7.8)
				Other	62 (34.4)
Financial situation	Satisfied	31 (17.2)	Contraceptive	None	81 (45)
	Intermediate	124 (68.9)		Hormonal	6 (3.3)
	Dissatisfied	25 (13.9)		None hormonal	93 (51.7)

The mean score of vaginismus diagnosis and its sub-areas are presented in Table 2. Also, the mean and standard deviation of the scores of sexual health different dimensions in women with vaginismus are shown in Table 3.

**Table 2:** The mean (SD) of gynecological examination, vaginismus score and its severity of 180 Iranian women with vaginismus

Variables	Mean (SD)	N (%)			
		Slight	Intermediate	Intense	Very intense
Gynecological examination	12.11 (2.70)	8 (4.4)	24 (13.3)	59 (32.8)	89 (49.4)
Physical suffering and inability to vaginal penetration (sub scale)	20.61 (2.80)	–	6 (3.3)	86 (47.8)	88 (48.9)
Inappropriate feeling in sex	9.72 (2.56)	16 (8.9)	70 (38.9)	71 (39.4)	23 (12.8)
Inadequate conditions in sexual intercourse	6.39 (1.43)	11 (6.1)	103 (57.2)	51 (28.3)	15 (8.3)
Fear feeling	22.71 (4.73)	2 (1.1)	74 (41.1)	82 (45.6)	22 (12.2)
Total score	59.43 (7.79)	–	19 (10.6)	134 (74.4)	27 (15)

**Table 3:** The mean (SD) of domains of sexual health of 180 Iranian women with vaginismus

Variables		Mean (SD)	Median
Depression		8.41 (5.71)	7
Stress		9.88 (5.04)	9
Anxiety		7.96 (5.07)	7
Fear of sex		23.46 (8.48)	23
Sexual self-efficacy		14.85 (5.39)	15
Sexual quality of life		67.30 (13.87)	67
Sexual satisfaction		75.06 (7.58)	75
Sexual function		16.43 (6.58)	18.10
Marital satisfaction		29.85 (4.14)	30
Sexual violence		2.99 (4.07)	1
Sexual intimacy		90.60 (13.63)	92
Sexual knowledge and attitude		117.16 (15.03)	119
Vaginal penetration cognition	Catastrophic and control cognition	46.54 (10.14)	48.5
	Positive cognition	13.18 (5.62)	13
	Self-image cognition	14.22 (3.09)	14.5

First, univariate linear regression was used, in which the dependent variable was the total score of the vaginismus diagnosis and independent variables were entered into the model one by one (Table 4). The variables of education, depression, stress, anxiety, fear of sex, sexual self-efficacy, quality of sexual life, sexual function, sexual violence, sexual intimacy, sexual knowledge and attitude, catastrophic cognition and control, positive cognition and mental image cognition were significantly associated with the variable of Vaginismus diagnosis. Then, in order to measure the vaginismus-related bio-psychosocial factors, multiple linear regression was used and significant variables of linear regressions were entered into the model. Initially, the variance inflation factor (VIF) was used to investigate Multicollinearity, but no significant correlation was observed between the variables of the final model (VIF > 10). Regression results showed that 45.5% of the variance of the total score of vaginismus diagnosis could be explained by these variables (R = 0.706, R^2^ = 0.498 and ADJ.R^2^ = 0.455).

**Table 4:** Univariate linear regression between total score of vaginismus and socio-demographic and sexual variables

Variables	B	P value	95% Confidence interval	
			Lower	Upper
Age	− 0.053	0.628	− 0.267	0.162
Age of partner	− 0.060	0.588	− 0.279	0.159
Duration of relationship	− 0.006	0.968	− 0.291	0.303
Education	− 2.013	**0.025**	− 3.770	− 0.258
Education of partner	− 0.224	0.836	− 2.364	1.916
Employment	− 1.291	0.313	− 3.807	1.226
Employment of partner	− 1.538	0.217	− 3.989	0.913
Marriage pattern	− 0.516	0.659	− 2.821	1.788
Financial situation	− 1.696	0.104	− 3.747	0.355
Depression	0.679	** < 0.001**	0.504	0.854
Stress	0.740	** < 0.001**	0.539	0.940
Anxiety	0.628	** < 0.001**	0.420	0.836
Fear of sex	0.434	** < 0.001**	0.314	0.554
Sexual self-efficacy	− 0.397	** < 0.001**	− 0.603	− 0.192
Sexual quality of life	0.255	** < 0.001**	0.281	0.330
Sexual satisfaction	− 0.011	0.887	− 0.163	0.141
Sexual function	− 0.418	** < 0.001**	− 0.582	− 0.254
Marital satisfaction	− 0.084	0.552	− 0.362	0.194
Sexual violence	0.636	** < 0.001**	0.369	0.903
Sexual intimacy	− 0.235	** < 0.001**	− 0.312	− 0.158
Sexual knowledge and attitude	− 0.168	** < 0.001**	− 0.241	− 0.096
Catastrophic and control cognition	0.379	** < 0.001**	0.280	0.478
Positive cognition	− 0.486	** < 0.001**	− 0.678	− 0.294
Self-image cognition	1.257	** < 0.001**	0.935	1.580

Bold is significant (p < 0.2); Estimated unstandardized regression coefficients with 95% Confidence intervals are mentioned in this table

Based on this multiple linear regression model, the variables of fear of sex (B = 0.141, P = 0.036), positive cognition (B = -0.197, P = 0.046) and self-image (B = 0.651, P = 0.001) in the psychological domain, and the variables of sexual intimacy (B = − 0.116, P = 0.021), quality of sexual life (B = 0.115, P = 0.002) and education (B = 2.129, P = 0.024) in the interpersonal domain of the bio-psychosocial model were the final predictors of vaginismus diagnosis score in women with vaginismus (Table 5). Accordingly, for each increase in the score of the fear of sex, self-image and quality of life, provided that all variables remain constant, the score of vaginismus diagnosis increased by 0.141, 0.651 and 0.115 respectively. For each increase in the person's educational grade, provided that all variables remain constant, vaginismus diagnosis score increased by 2.129 points on average. Finally, for each increase in the score of positive cognition of vaginal penetration and sexual intimacy, provided that all variables remain constant, vaginismus diagnosis score decreased by 0.197 and 0.116 respectively.

**Table 5:** Multivariate linear regression between total score of vaginismus and socio-demographic and sexual variables

Biopsychosocial factor		B	SE	P value	95% Confidence Interval	
					Lower	Upper
(Constant)		38.678	6.828	** < 0.001**	25.195	52.161
Biological domain	Sexual function	− 0.005	0.082	0.954	− 0.167	0.158
Psychological domain	Depression	0.008	0.192	0.968	− 0.372	0.387
	Stress	0.112	0.203	0.581	− 0.288	0.513
	Anxiety	0.114	0.157	0.470	− 0.196	0.423
	Fear of sex	0.141	0.066	**0.036**	0.010	0.272
	Sexual self-efficacy	0.022	0.104	0.830	− 0.182	0.227
	Sexual knowledge and attitude	0.019	0.045	0.679	− 0.071	0.109
	Catastrophic and control cognition	0.024	0.065	0.714	− 0.104	0.152
	Positive cognition	− 0.197	0.098	**0.046**	− 0.390	− 0.004
	Self-image cognition	0.651	0.187	**0.001**	0.281	1.021
Interpersonal domain	Sexual violence	0.019	0.125	0.879	− 0.228	0.266
	Sexual intimacy	− 0.116	0.050	**0.021**	− 0.214	− 0.018
	Sexual quality of life	0.115	0.037	**0.002**	0.043	0.187
	Education	2.129	0.935	**0.024**	0.282	3.976

Bold is significant (p < 0.2); Estimated unstandardized regression coefficients with 95% Confidence intervals are mentioned in this table

## Discussion

The aim of this study was to investigate the vaginismus-related bio-psychological factors. Various physical, psychological, social and cultural factors can contribute to the development of vaginismus. Therefore, this disorder is considered to be a multidimensional disorder [42]. The results of the present study showed that the variables of fear of sex, positive cognition and self-image in the psychological domain of the bio-psychosocial model were predictors of vaginismus diagnosis score. As such, any increase in the score of the fear of sex increased the score of vaginismus. Patients with vaginismus often experience a form of avoidance phobia, fear, and pain accompanied by involuntary muscle contraction [43]. Indeed, this phobia occurs before the onset of vaginismus and may be accompanied by negative related cognitions and expectations [44]. Women with vaginismus will usually experience severe anxiety and stress with any penetration, which will lead to severe avoidant behaviors such as pushing, squeezing their legs together and screaming. Interestingly, only a relatively small number of women report a history of sexual or other trauma and, thus, pre-existing sexual trauma does not conclusively predict these conditions [45].

Additionally, misconceptions about the genitals (e.g. a very tight or different vagina and an oversized penis) seem to provide the basis for avoiding sex in women with vaginismus [46]. The results of the present study are in line with the behavioral model of vaginismus (fear avoidance model). In this model, fear is a conditional response to avoid all cases of vaginal penetration that can prevent treatment. This is because women with vaginismus can usually experience vaginal penetration when their fear has subsided [30]. Accordingly, future research can focus on recognizing the stimuli that are the main cause of fear in vaginal penetration, as well as various methods for reducing anxiety and stress in the treatment of vaginismus.

The results of the present study indicated that in the psychosocial domain, any increase in negative self-image increased the score of vaginismus. Body image and genital self-image refer to a person's feelings and beliefs about their body and genitals, both of which are involved during sexual activity [47]. Comparing the cognitions of vaginal penetration in women with and without vaginismus, Klaassen et al. found that women with vaginismus had a more negative self-image and reported some incompatibilities about their genitals during vaginal penetration [12]. Barnes et al. concluded that women with vaginismus did not have a clear understanding of vaginal muscle tone and could not distinguish between relaxation and muscle spasm, and were unaware of their ability to change vaginal muscle tone voluntarily [48].

On the other hand, in the present study, vaginismus score decreased for each increase in the score of positive cognition. A woman's positive attitudes towards sex may strengthen her sense of self-efficacy so that she can cope with her pain [49]. This is done in such a way that a woman's attention is distracted from vulvovaginal pain so that she can focus her attention on the pleasurable and satisfying aspects of sexual life [50]. Evidence suggests that high levels of sexual information increases positive attitudes towards vaginal penetration, thereby modifying catastrophic cognitions of vaginal penetration [51].

Moreover, the present study showed that the variables of sexual intimacy, quality of sexual life and education in the interpersonal domain of the bio-psychosocial model were predictors of vaginismus diagnosis score. Thus, any increase in sexual intimacy score decreased the vaginismus score. In line with the results of the present study, Alizadeh et al. revealed in 2019 that intimacy with spouse, sexual satisfaction and marital satisfaction were significantly associated with genito-pelvic pain/penetration disorder (GPPPD) [8]. Clinically speaking, it has been repeatedly observed that sexual problems may be the cause or result of dysfunctional or unfriendly relationships [52]. Based on recent studies, one of the causes of vaginismus in women is the fear of intimacy with the husband, which prevents intimacy, close relationship and intercourse [18]. As mentioned earlier, sexual self-expression and talking about sexual issues, desires, and sexual preferences are among the interpersonal factors that are considered to be important in establishing a desirable sexual relationship. By contrast, when couples do not talk about sexual issues, they will enjoy less which leads to more sexual problems. The reason is that an issue will not be resolved and may lead to sexual dissatisfaction unless it is addressed by the couples [53]. On the other hand, people who express more positive emotions, feel higher levels of sexual satisfaction and intimacy with their spouses, experience less stress and are less likely to divorce as they can reconcile their marital conflicts more effectively [54].

Based on the results of the present study, any increase in a person's education can increase the score of vaginismus. In line with the present research, recent studies have shown that vaginismus is more prevalent in educated women with high socioeconomic status [11]. Farnam et al. found that women with vaginismus, despite being educated, had less sexual knowledge at the time of marriage than women of the control group [21]. In fact, higher levels of education do not mean having higher sexual knowledge, and education does not prevent cognitive distortions and misconceptions about sex [10]. According to recent studies, there is more adaptation to the sexual problem of vaginismus in educated couples, which can prolong the problem [55]. By contrast, in couples with lower levels of education and socioeconomic status, vaginal penetration occurs earlier that is due to the husband's insistence and stronger influence. Accordingly, these women will experience other sexual problems (e.g. discomfort during urination and defecation) after vaginal penetration, which are caused by the husband's violent attempt to perform vaginal penetration [56].

According to the results of the present study, any increase in the quality of sexual life score increased the score of vaginismus. In a study in Turkey, Doğan et al. showed that vaginismus affected the quality of life and the level of sexual satisfaction in couples [57]. Based on the clinical experience, some sexual partners may accept their partner's avoidant behaviors and try to improve the quality of their sexual life without vaginal penetration. The reason is that they are concerned about the pain of their partner and have avoidant behaviors for having the vaginal penetration. The admirable perception and "noble" behavior of such partners paradoxically leads to the stability of their lives and improves the quality of their sexual life [45]. As such, most couples who because of vaginismus do not have vaginal penetration may surprisingly experience a satisfactory sex without any vaginal penetration [21].

One of the strengths of the present study was the use of a powerful model for predicting vaginismus-related factors in the three biological, psychological and interpersonal domains. Another strength is the use of a special questionnaire for measuring vaginismus in addition to the confirmation of the infection by a specialist. One of the limitations of the present study was the lack of a suitable control group to compare with the intervention group so as to confirm the accuracy of the research results more decisively and generalize the results to other societies. Future studies can predict all aspects of sexual health of women with vaginismus in the form of structural equation model and compare them with the control group (those without vaginismus).

The results of the present study also emphasize the importance of using a bio-psychosocial approach when investigating sexual pain disorders. In the present study, biological domain cannot always be emerged as a strong predictor. Instead, psychological and interpersonal variables were the more prominent predictors of vaginismus. Evaluation of women’s psychosocial context may provide us with a deeper understanding of vaginismus and its impact on both women and theirs partners. The need for a bio-psychosocial approach and the significance of considering interpersonal and psychological contexts in investigating sexual problems has been noted in previous literature [58, 59]. This research considered the role of bio-psychosocial variables which may show themselves in the form of pain disorders. Therefore, designing interventions, this model can be used for the treatment of vaginismus in women, especially in the psychological and interpersonal domains. As a rule, after identifying the predictors, the interventions should include methods for reducing the identified factors. For example, fear and anxiety reducing methods should be adopted to eliminate vaginismus.

## Conclusion

Various physical, psychological, social and cultural factors can influence the development of vaginismus. Therefore, this disorder is considered to be a multidimensional disorder. The results of the present study revealed that the variables of fear of sex, positive cognition and negative self-image, sexual intimacy, quality of sexual life and education from the bio-psychosocial model were the final predictors of vaginismus diagnosis score. The present study showed that the bio-psychosocial model is perfectly able to predict the status of women with vaginismus.

## Data Availability

The datasets of the present study are available from the corresponding author on reasonable request.

## Abbreviations

DSM-IV: Diagnostic and Statistical Manual of Mental Disorders 4th edition
DASS: Depression, Anxiety and Stress Scale
VPCQ: Vaginal Penetration Cognition Questionnaire
ICC: Intra class correlation coefficient
SEM: Standard error of measurement
MDC: Minimum detectable changes
VIF: Variance inflation factor
GPPPD: Genito-pelvic pain/penetration disorder
